# Pandemic Policy and Life Satisfaction in Europe

**DOI:** 10.1111/roiw.12554

**Published:** 2021-10-29

**Authors:** Andrew E. Clark, Anthony Lepinteur

**Affiliations:** ^1^ Paris School of Economics—CNRS; ^2^ University of Luxembourg

**Keywords:** COVID‐19, life satisfaction, policy stringency, economic support

## Abstract

We use data from the COME‐HERE longitudinal survey collected by the University of Luxembourg to assess the effects of the policy responses to the COVID‐19 pandemic on life satisfaction in France, Germany, Italy, Spain and Sweden over the course of 2020. Policy responses are measured by the Stringency Index and the Economic Support Index from the Blavatnik School of Government. Stringency is systematically associated with lower life satisfaction, controlling for the intensity of the pandemic itself. This stringency effect is larger for women, those with weak ties to the labor market, and in richer households. The effect of the Economic Support is never statistically different from zero.

## 1 Introduction

The COVID‐19 pandemic has drastically changed our lives. These have for example become much more sedentary (with less physical activity and more screen time) everywhere in the world (Hu *et al*., 2020; Kumari *et al*., 2020; Medrano *et al*., 2020; Giuntella *et al*., 2021). On the labor market, unemployment and job insecurity have been on the rise, while working time has fallen (Adams‐Prassl *et al*., 2020; Bottan *et al*., 2020; Guven *et al*., 2020; Beland *et al*., 2021). Brewer and Gardiner (2020) use the Resolution Foundation’s Coronavirus Survey, a cross‐section dataset of 6,000 UK adults in early May 2020, to show that the probability of reporting lower household income has risen. In Belot *et al*. (2021), cross‐section data from China, Japan, South Korea, Italy, the UK and the US in April 2020 (around 1,000 respondents per country) reveals that the youngest were more likely to experience drops in household income.

Aknin *et al*. (2021) review the growing literature on the consequences of living through the COVID‐19 pandemic on mental health and subjective well‐being. They conclude that the pandemic has triggered a rise in mental‐health issues, while the evidence on cognitive well‐being measures is more nuanced. Although overall 2020 well‐being trends certainly partly reflect the spread of COVID‐19 itself, we here focus on the well‐being consequences of governmental policy responses. Using the Gross National Happiness Index derived from Twitter, Greyling *et al*. (2021) uncover a negative and significant well‐being effect of lockdown measures in New Zealand, Australia and South Africa. Using a combination of difference‐in‐differences regressions and regression‐discontinuity designs, Brodeur *et al*. (2021) show that the lockdown measures in Western Europe had a negative impact on a number of aspects of well‐being, as measured by topic searches in Google Trends. The effect in the US, however, is positive. The difference between Western Europe and the US is argued to reflect timing of the measures, with the US locking down later (and the well‐being effect of lockdown also being positive in the “later‐lockdown” countries in Europe: Ireland, Portugal and the UK).

Some work has appealed to individual‐level data. Fancourt *et al*. (2020) consider lockdown and mental health in the UK, using a longitudinal observational study (the UCL COVID‐19 Social Study). They find that depression and anxiety levels fell during the weeks following the lockdown introduction. Based on the high‐frequency USC Understanding Coronavirus in America Study, Banks *et al*. (2021) show a reduction in the prevalence of anxiety, depression and other mental‐health measures such as self‐perceived stress following the lockdown of April 2020. On the contrary, Sibley *et al*. (2020) find worse mental health after the introduction of lockdown in panel data from New Zealand. Combining the German Job Search Panel (a longitudinal survey of employed job seekers registered at the Federal Employment Agency) and an event‐study design, Schmidtke *et al*. (2021) find that the first federal lockdown in Germany during March and April 2020 reduced life satisfaction, affective well‐being and mental health. To our knowledge, there has not been work explicitly relating subjective well‐being to the changing government pandemic policy responses within different countries using panel data throughout 2020. This is what we do here, exploiting the changes in governments’ pandemic policy responses over time across five European countries. We consider both the stringency of lockdown measures and the economic support provided by governments, which we match to life‐satisfaction scores in France, Germany, Italy, Spain, and Sweden from a large panel survey covering over 8,000 individuals.

Controlling for the evolution of the pandemic itself (via the 4‐week average number of daily deaths), our panel analysis reveals that more‐stringent policies significantly reduce life satisfaction. In line with the literature suggesting rising gender inequality during the pandemic, this drop in life satisfaction from confinement is larger for women. It is also larger for respondents with the weakest ties to the labor market, and for those with a relatively high income. The former is consistent with greater feelings of job insecurity caused by the labor‐market disruption from lockdown, while the latter may reflect the restrictions on certain types of leisure consumption that are more prevalent among the better‐off (for example, international tourism, restaurants and theatre).

On the contrary, there is no evidence of a link between the generosity of economic support and life satisfaction. This insignificance could indeed be read as showing that government economic support did not make any mark on subjective well‐being. However, the income‐support schemes implemented throughout 2020 did not evolve at random. In particular, they were designed to increase household incomes (Clark *et al*., 2021b) relative to the case with no intervention. By compensating for the income losses caused by lockdown stringency, economic support helped keep individuals’ economic resources at their non‐lockdown level. Our regressions then compare “normal” income to lockdown income plus compensation; with these two being fairly similar by design we may not expect to uncover a significant relationship with life satisfaction.

The remainder of the paper is structured as follows. Section 2 describes the data and the empirical strategy. Section 3 then estimates the effects of governmental policy responses to COVID‐19 on life satisfaction, and identifies those who have been more‐strongly affected. Last, Section 4 concludes.

## 2 Data and Empirical Strategy

### 2.1 Data

The data we use here comes from the ongoing COME‐HERE (COVID‐19, MEntal HEalth, REsilience and Self‐regulation) survey collected by the University of Luxembourg. The survey was conducted with Qualtrics to produce representative samples of adults (aged 18 or over) in France, Germany, Italy, Spain and Sweden.^1^ Respondents were asked to complete an on‐line questionnaire that takes approximately 20 minutes. The survey collects information at both the individual and household levels, and is longitudinal. The first four waves of the COME‐HERE survey were conducted around late April, early June, early August, and late November 2020. At least four more waves are planned to take place in 2021.

More than 8,000 individuals responded to the first survey wave, and were then invited to take part in the subsequent waves. Over 80 percent of Wave One respondents participated in at least one other survey wave, with 45 percent participating in all four. The survey collects detailed information on individuals’ living conditions and mental health during the pandemic, as well as identifying recent changes and events in their lives. The survey also includes standard sociodemographic characteristics such as age, gender, education, labor‐force status, and country and region of residence.

In each survey round, respondents replied to the following life‐satisfaction question “*Overall, in the past week, how satisfied have you been with your life?*” using a standard 11‐point Likert scale. Life satisfaction is one of the most common cognitive measures of well‐being. An extensive empirical literature has suggested that life satisfaction, and well‐being questions more generally, constitutes a valid measure of individual utility (see Clark, 2016, for a detailed review of this literature). Life satisfaction questions allow each individual to put their own weights on each dimension of their lives that they consider as relevant to produce an overall summary score. While well‐being questions are subjective, their validity has been addressed by showing that their responses are correlated with physiological expressions of emotions and brain activity (Urry *et al*., 2004) as well as with future behaviors, such as marital break‐up (Guven *et al*., 2012), job quits (Clark, 2001), productivity (Oswald *et al*., 2015) and voting (Liberini *et al*., 2017; Ward, 2020). Subjective well‐being measures then contain useful information, in that one person who says that they are less satisfied with their job (for example) is more likely to quit it than another person with a higher satisfaction score. This cross‐sectional correlation between well‐being and behavior underscores that the former is informative about individuals’ unobserved real quality of life: at least to some extent, people mean what they say. Were subjective scores to be incomparable across individuals, no such relationship would be found.

**Figure 1:**
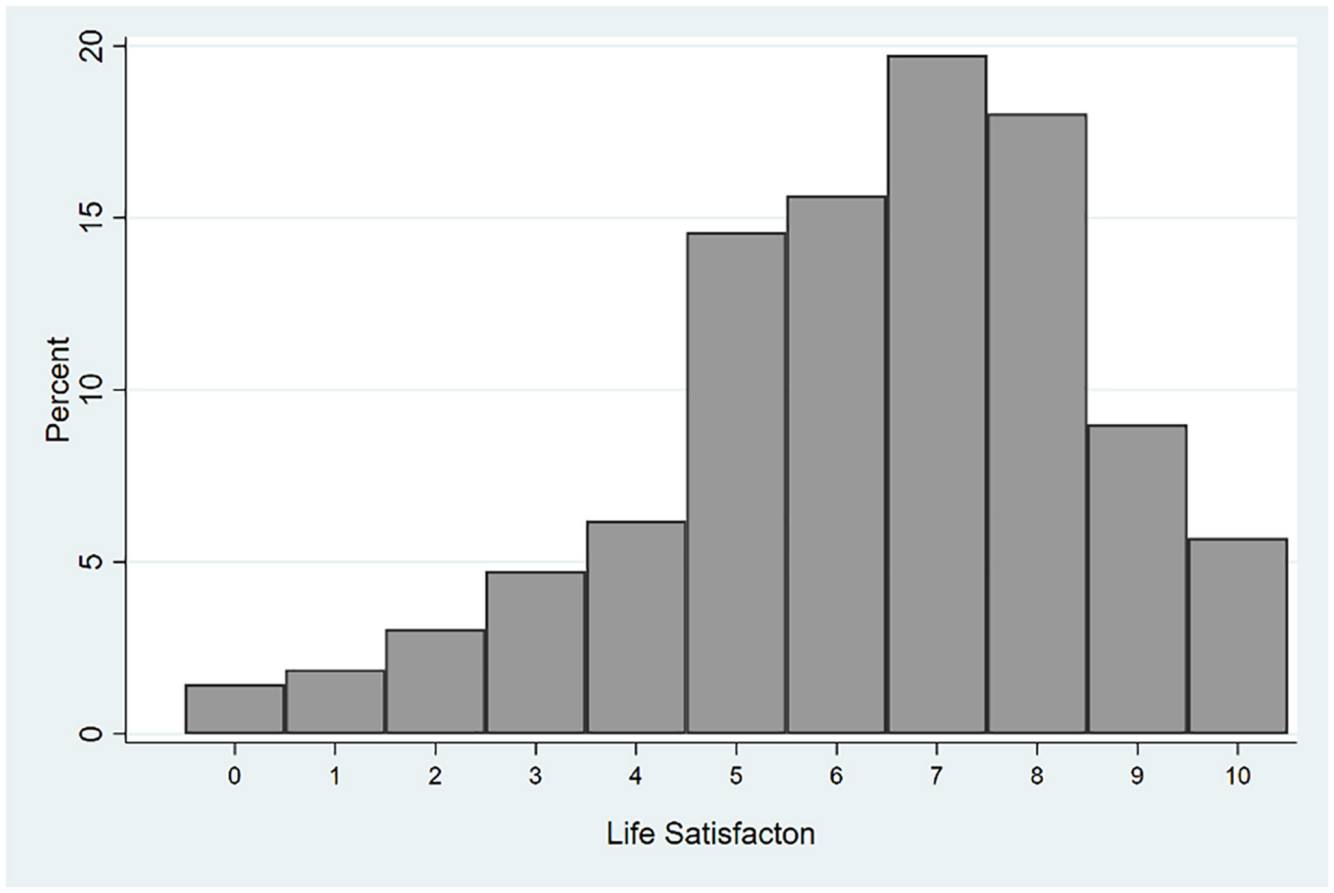
The Distribution of Life Satisfaction in our Estimation Sample *Note*: These data refer to the estimation sample in the four 2020 waves of COME‐HERE survey data.

The distribution of life satisfaction in the estimation sample that we will use below, the four 2020 waves of COME‐HERE, appears in Figure 1. There is left‐skew, as is often found in subjective variables. The mean level of life satisfaction is 6.3 on the 0–10 scale, and the modal response is 7.

### 2.2 Empirical Strategy

We estimate the following equation via OLS with individual fixed‐effects:

(1) $$LS_{\mathrm{ijt}}=\alpha SI_{\mathrm{jt}}+\beta ESI_{\mathrm{jt}}+\gamma COVID_{\mathrm{jt}}+\delta X_{\mathrm{ijt}}+\mu_{i}+\lambda_{t}+\varepsilon_{\mathrm{ijt}}.$$

Here *LS_ijt_* is the life satisfaction of respondent *i* living in country *j* at time *t*. *SI_jt_* and *ESI_jt_* are, respectively, the Stringency Index and the Economic Support Index in country *j* at time *t*, which form part of the Oxford COVID‐19 Government Response Tracker produced by the Blavatnik School of Government at the University of Oxford. Over one hundred students and staff members of the University of Oxford from every part of the world collect data from public sources to produce indices measuring policy responses to COVID‐19 at the national level that are updated on a daily basis. The Stringency Index is composed of the nine following sub‐indices, measuring various aspects of containment policies: “school closing,” “workplace closing,” “cancellation of public events,” “restriction on gathering,” “public transport closing,” “stay‐at‐home requirements,” “restriction on internal movement,” “restriction on international travel” and “public information campaign”. The Economic Support Index instead has only two components: “income support” and “debt relief.” The first measures the extent to which governments provide their citizens with direct cash payments, universal basic income, or income support for those who lost their job or cannot work; the second pertains to governmental decisions to freeze the financial obligations of households (such as loan repayments).^2^

Both *SI* and *ESI* are rescaled so that they range from 0 to 100. A higher value of *SI* corresponds to a more‐stringent country lockdown‐style policy response to COVID‐19. Equally, higher *ESI* scores reflect the country replacing a higher percentage of lost earnings and/or providing greater debt/contract relief in attempting to counterbalance the adverse economic effects of COVID‐19 on individuals. As explained by the data producers (Hale *et al*., 2020), these indices do not measure the effectiveness of a government’s response in terms of outcomes but are rather synthetic measures of the intensity of government policy that can be compared cross‐country and over time. In our main regressions, *SI_jt_* and *ESI_jt_* are the average index values over the two weeks prior to the interview date, and are standardised to have means of zero and standard deviations of one. We will show in the robustness checks that our results are similar when we calculate the index values over different time horizons.^3^

We do not have a strong prior about the sign of *α*. Containment policies aim to limit the spread of the virus, with public‐health benefits that likely contribute to well‐being. However, at the same time they impose more sedentary lifestyles, restrict social interaction, and disrupt the economy. The net effect of *β* is also ambiguous. If the economic support provided by governments compensates the income losses produced by the pandemic, *β* will reflect the net effect of lower income that is then (partly) compensated. If income‐support schemes fully compensate for losses, the net effect of *β* will depend on the relative importance of income losses and gains on well‐being. If these are equal, *β* would then be zero; if on the contrary losses weigh more than gains (as in Boyce *et al*., 2013; De Neve *et al*., 2018), *β* will be negative.

The *SI_jt_* and *ESI_jt_* policy variables are of course not random, and reflect the spread of COVID‐19. With no other controls, the estimated coefficients on *SI_jt_* and *ESI_jt_* would be confounded by the omitted variable of the evolution of COVID‐19 itself. We attenuate this bias by controlling for the extent of the pandemic, *COVID_jt_*. Plausible candidates for *COVID_jt_* are the total and daily number of COVID‐19 cases and deaths (averaged over the previous two or four weeks). It is difficult to include all of these variables at the same time as they are very strongly correlated, and we control for only one measure in our main empirical model. The preferred *COVID_jt_* measure is identified in Tables A1 and A2 as that which best fits *SI_jt_* and *ESI_jt_* respectively, over the interview dates of the COME‐HERE survey in 2020. In Table A1, for *SI_jt_*, this is the average number of daily deaths over four weeks (*R* ^2^ = 0.686); for *ESI_jt_* in Table A2 it is the four‐week average of the cumulative number of deaths since the beginning of the pandemic (*R* ^2^ = 0.320). We will use four‐week average daily deaths as our *COVID_jt_* measure due to the better quality of the fit. The results are qualitatively similar when we use any of the other COVID‐19 measures.

The vector *X_it_* includes standard individual characteristics (age and its square, the log of equivalised monthly household disposable PPP‐adjusted income in January 2020, and dummies for gender, partnership status, education, labor‐force status, and country of residence). We control for macro‐trends and individual time‐invariant heterogeneity by introducing respectively wave fixed‐effects *λ_t_* and individual fixed‐effects *μ_i_*.^4^ In our panel estimations, all of the *X_it_* variables will be dropped apart from labor‐force status. Standard errors are clustered at the *SI_jt_* * *ESI_jt_* level. We will also present a number of robustness checks to show that our conclusions hold with different versions of our main specification.

We consider the sample of COME‐HERE respondents who were present in at least two out of the four 2020 survey waves, and who provided valid information on life satisfaction and the socio‐demographic variables. This sample consists of 20,337 observations (on 6,039 individuals); the associated descriptive statistics appear in Table 1. French, German, Italian and Spanish respondents make up a little over 20 percent of the sample each, while 12 percent of the observations come from Swedish respondents. In terms of the wave structure, 30 percent of the observations are from Wave One, and the remainder are fairly equally distributed across the three remaining waves. Just under half of the sample observations come from women and the high‐educated (i.e., those with a diploma from post‐secondary education). As with all panel surveys, there is some attrition. Although we do not use weights in our main specification, we will show in the robustness checks that we obtain similar results when we address non‐random attrition via Inverse‐Probability Weights.

**TABLE 1:** Descriptive Statistics

	Mean	SD	Min	Max
Life satisfaction	6.34	2.20	0	10
*OxCGRT measures*				
Stringency Index	69.2	13.3	46.3	93.5
Economic Support Index	67.6	19.1	29.7	100
*COVID measure:*				
Average daily deaths/100,000 inhabitants (4 weeks average)	0.399	0.391	0	1.26
*Individual characteristics:*				
Log equivalent household income (Jan 2020—in PPP)	7.33	0.678	5.34	9.54
Family size	3.07	1.36	1	10
Age	50.0	15.96	18	93
Female	0.482		0	1
Partnered	0.611		0	1
Primary education	0.190		0	1
Secondary education	0.381		0	1
Tertiary education	0.429		0	1
In full‐time employment	0.465		0	1
In part‐time employment	0.095		0	1
In marginal employment	0.014		0	1
Not in employment	0.425		0	1
Key‐sector employee (Jan 2020)	0.250		0	1
Other‐sector employee (Jan 2020)	0.323		0	1
*Wave*				
W1: April 2020	0.297		0	1
W2: June 2020	0.212		0	1
W3: August 2020	0.244		0	1
W4: November 2020	0.247		0	1
*Country of residence*				
France	0.226		0	1
Germany	0.215		0	1
Italy	0.215		0	1
Spain	0.222		0	1
Sweden	0.121		0	1
*Observations*	*20,337*			
*Individuals*	*6,039*			

*Note:* These numbers refer to respondents from the four 2020 waves of the COME‐HERE survey.

**Figure 2:**
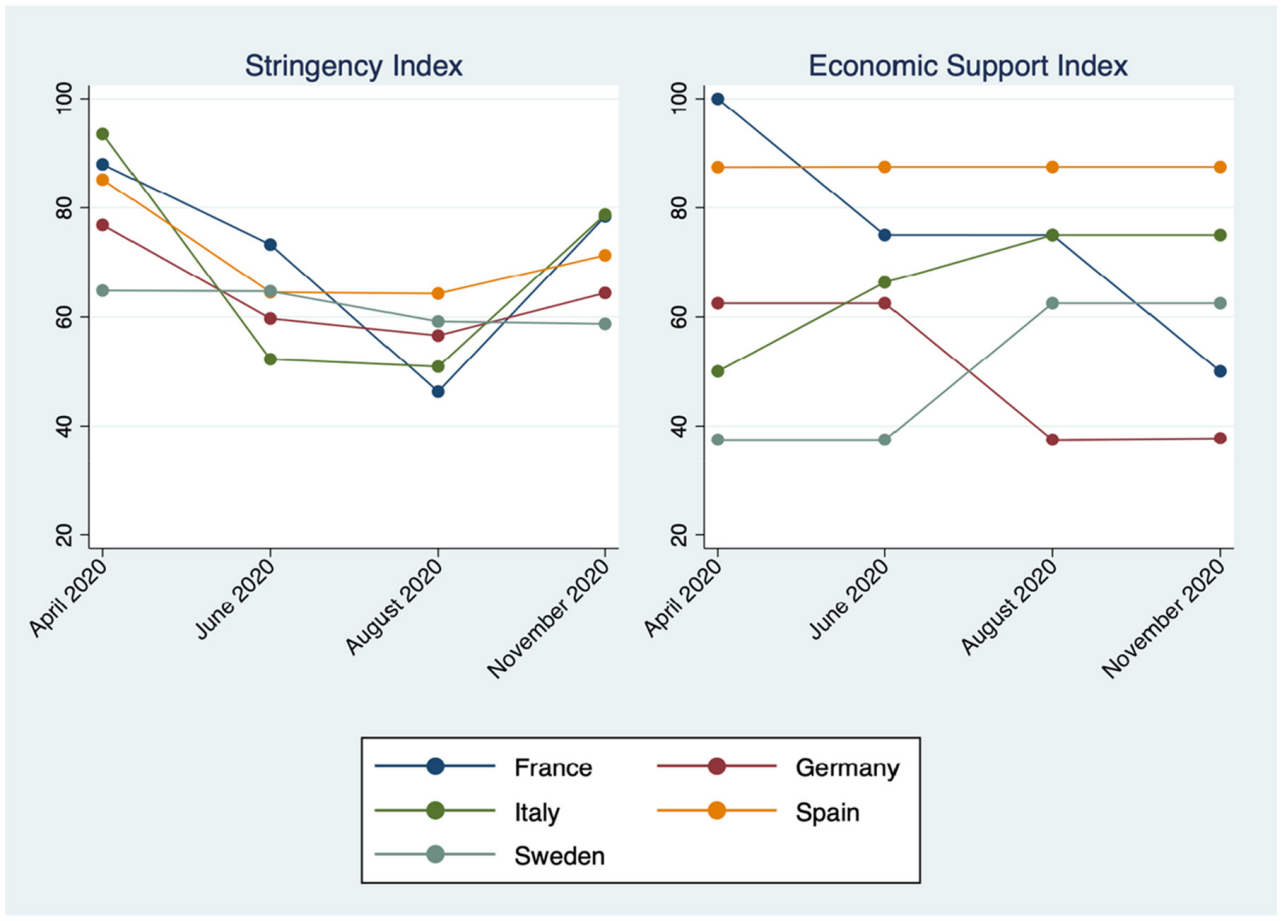
The Stringency and Economic Support Indices by country and wave *Notes*: The dots refer to the average values of the indices across the various interview dates in each of the four 2020 waves of the COME‐HERE survey, by country of residence. The index values come from the Oxford COVID‐19 Government Response Tracker of the Blavatnik School of Government.

Figure 2 depicts the evolution of the Stringency and Economic Support Indices across the five COME‐HERE countries over April to November 2020. At each of the four waves, the dots refer to the average values of the indices across the various interview dates at that wave (where the value taken by the indices on any day is itself an average over the two weeks prior to that day). Stringency is U‐shaped in almost every country: the index fell after the first COVID‐19 wave and the release of the first lockdowns in Europe (from April to August) but then rose with the second COVID‐19 wave and the new series of lockdowns (from August to November). The only country with a different pattern is Sweden, where no lockdowns were introduced: here the Stringency Index is on the contrary fairly flat over 2020, taking on the lowest average figure among the COME‐HERE countries.

On the right‐hand side of Figure 2 the pattern for the Economic Support Index across countries is more nuanced. This Index remained high and stable in Spain throughout the sample period, while it rose over time in Italy and Sweden but fell in France and Germany. This reflects the key economic responses summarised by the International Monetary Fund’s policy tracker (see www.imf.org/en/Topics/imf‐and‐covid19/Policy‐Responses‐to‐COVID‐19). The first economic support measures in Spain appeared as early as the beginning of March, with an overall budget of 47 Billion Euros (4.2 percent of Spanish GDP) being agreed by the government over the course of 2020. These measures included easier access to unemployment benefits, higher sick pay for COVID‐19 infected workers, support for the self‐employed who lost work, a new means‐tested minimum‐income scheme, and subsidising new rental programs for vulnerable renters. The governments in the other COME‐HERE countries implemented somewhat‐similar economic support schemes. In Italy, an emergency package of around 25 Billion Euros (1.6 percent of Italian GDP) was announced in March 2020. In line with the evolution of the Economic Support Index in Figure 2, the government adopted additional packages of fiscal measures during 2020 to support families, the health‐care system and businesses. Around 100 Billion Euros had been devoted to support packages by the Italian government by the end of 2020. As in Italy, the Swedish government gradually increased its support to the families and businesses during the course of 2020. Around 16 percent of Swedish GDP was allocated to capital injections, liquidity support and family subsidies. Similar packages were also adopted by the French and German governments early in 2020, although these became more targeted over time.

## 3 Pandemic Policy and Life Satisfaction

### 3.1 Main Results

Table 2 lists the regression results from the estimation of Equation (1). Columns (1) and (2) introduce the Stringency Index and Economic Support Index separately in an equation with no other controls: the former attracts a negative and very‐significant estimate but there is no significant correlation with the latter.^5^ We then introduce both at the same time in column (3): although the two indices are positively correlated, with a Pearson’s correlation coefficient of 0.18, this produces only very little change in the estimated Index coefficients.^6^ Column (4) then addresses any confounding effect from the spread of COVID‐19 itself by controlling for the 4‐week average number of daily deaths. This turns out to have only a slight attenuation effect on the estimate for the Stringency Index, which remains significant; there continues to be no evidence of a relationship between the Economic Support Index and life satisfaction in our five countries over the course of 2020. The 4‐week average number of daily deaths attracts a negative, but insignificant, estimated coefficient.^7^

**TABLE 2:** Pandemic Policy and Life Satisfaction—Pooled and Panel Results

	Life Satisfaction (Standardised)					
	(1)	(2)	(3)	(4)	(5)	(6)
Stringency Index	−0.056***		−0.057***	−0.051**	−0.050**	−0.046**
	(0.019)		(0.020)	(0.021)	(0.020)	(0.020)
Economic Support Index		0.002	−0.005	−0.007	−0.008	−0.010
		(0.021)	(0.017)	(0.017)	(0.017)	(0.015)
Average daily deaths/100,000 inhabitants				−0.018	−0.026	−0.017
(4‐week average)				(0.017)	(0.017)	(0.014)
*Wave and country FE*	Yes	Yes	Yes	Yes	Yes	Yes
*Controls*	No	No	No	No	Yes	Yes
*Individual FE*	No	No	No	No	No	Yes

*Notes:* These are linear regressions. The sample here is respondents from the four 2020 waves of the COME‐HERE survey; there are 20337 observations in each column. The Stringency Index, Economic Support Index and average daily deaths variable are all standardised over the estimation sample. Standard errors in parentheses are clustered at the Stringency Index*Economic Support Index level. The cross‐sectional controls are age and its square, gender, family size and relationship status (all measured at Wave 1), the log of equivalent household disposable income in January 2020 in PPP, and dummies for education and labor‐force status. In the panel regression in column (6), we only retain labor‐force status. *, **, and *** respectively indicate significance levels of 10%, 5% and 1%.

Column (5) introduces controls for individual heterogeneity, via the variables in the *X_it_* vector. The estimates for the Stringency Index and Economic Support Index are unsurprisingly unaffected, as there is little reason to believe that changes in individual characteristics within a country over a short time period would be correlated with the government’s COVID‐19 policy responses. We last include individual fixed effects in column (6), which will reflect, for example, differences in the use of the life‐satisfaction scale. In these regressions, we do not include sex, education, marital status, or income in January 2020, as these do not change (or change only very little) over the seven‐month period. The introduction of individual fixed effects does not affect our estimates either: a one standard‐deviation higher Stringency Index is estimated to reduce life satisfaction by 0.05 of a standard deviation, while there continues to be no relationship with the Economic Support Index. The negative relationship between life satisfaction and the Stringency Index is in line with Brodeur *et al*. (2021). The effect of a one‐standard deviation increase in the Stringency Index in our data is sizeable, and equivalent in the cross‐section results of column (5) to one‐third of the coefficient on partnership, and not much less than the coefficient on tertiary education. The effect size is similar to that found by Adams‐Prassl *et al*. (2021) in their analysis of US data in March‐May 2020, where lockdown in the State of residence reduced the WHO‐5 mental‐health measure by 0.048 of a standard deviation.

During the pandemic, policy makers had to evaluate the costs and benefits of stringency policies such stay‐at‐home orders. From a life‐satisfaction perspective, our results suggest a net negative impact of policy stringency, even though these were designed to limit the spread of the virus. The well‐being effect of an increase of one standard deviation in the Stringency Index is three times that of a one standard‐deviation rise in the average number of daily deaths. Even though the latter estimate is not significant in the current analysis, it is of interest to calculate the implied trade‐off between stringency and mortality in our life‐satisfaction results.^8^

One standard deviation of mortality in our sample is four deaths per million per day. One month of higher stringency is thus “worth it” in well‐being terms if it saves three times four deaths per million per day for 30 days: in a country the size of the UK (with 66 million inhabitants), this figure is 23,760. Layard *et al*. (2020), in their well‐being evaluation of optimal lockdown policy in the UK, make the assumption that one month of lockdown would prevent 35,000 deaths. If a standard deviation of stringency is at least equal to two‐thirds of the stringency of a lockdown, then we suggest that stringency has raised societal well‐being.

The finding of no significant relationship between life satisfaction and the Economic Support Index is striking: Does this reflect the inadequacy of economic support programmes? This may seem unlikely, as we know that this governmental economic support reduced inequality and poverty (Clark *et al*., 2021a; Menta, 2021) and increased household incomes (Clark *et al*., 2021b) in the COME‐HERE survey countries. We believe that a more‐convincing explanation is that of an omitted variable. The Economic Support Index is higher exactly when (unobserved) economic needs due to reduced incomes were greater. With these needs reducing life satisfaction and causing higher values of the Support Index, our estimated coefficient on the latter in a life‐satisfaction equation is biased downwards. As such, economic support programs did very likely work, but we cannot see this in our data as we would need to compare different index values while holding (unobserved) needs constant.

### 3.2 Robustness Checks

Our baseline estimates are based on the average Stringency and Economic Support Index values over the two weeks prior to the interview date. However, some policies may take time to be effective, which could explain why we find no effect for the Economic Support Index. It can on the contrary also be argued that individuals adapt very fast to policy changes, making more recent index values more salient. We explore these possibilities in the first four columns of Table A4, where we look at the associations between life satisfaction and four new values of the indices. We consider the value of the indices at the exact day of the interview, and the average indices over the one‐, three‐ and four‐week periods prior to the interview date. In line with the baseline estimates, the Stringency Index always attracts a negative significant estimated coefficient, while that on the Economic Support Index is always statistically insignificant. Although the ESI coefficients are not significantly different from each other across the four columns, we can see that they become more positive as the time frame becomes longer: the economic support provided by governments may then well take time to become effective.

We now turn to attrition in our sample. While the resulting bias is often suggested to be limited (Fitzgerald and Gottschalk, 1998; Neumark and Kawaguchi, 2004; Cheng and Trivedi, 2015), we formally check whether this is also the case with our estimation sample in columns (5) and (6) of Table A4, where we respectively estimate our baseline regressions using the cross‐sectional and longitudinal weights from the COME‐HERE survey. The estimated coefficients are only little changed by these weights, so that attrition does not affect our conclusions.

### 3.3 Heterogeneity

Table 2 revealed the *average* effects of the COVID‐19 policy responses on life satisfaction. We may however believe that these are stronger for certain types of respondents. Table 3 thus shows the estimates from an augmented version of Equation (1) including interaction terms. To avoid endogeneity issues, this heterogeneity analysis appeals only to time‐invariant or pre‐COVID 19 characteristics. All of the regressions in Table 3 are panel, including individual fixed effects.

**TABLE 3:** Pandemic Policy and Life Satisfaction—Individual Heterogeneity Analysis: Panel Results

	Life Satisfaction (Standardised)				
	(1)	(2)	(3)	(4)	(5)
Stringency Index (*SI*)	−0.038*	−0.046**	−0.048**	−0.116***	−0.036*
	(0.020)	(0.020)	(0.020)	(0.010)	(0.020)
*SI interacted with*					
Female	−0.016**				
	(0.007)				
Tertiary education		0.002			
		(0.008)			
Partnered			0.004		
			(0.006)		
Retired				0.017*	
				(0.010)	
Key‐sector employee				0.020**	
				(0.010)	
Other‐sector employee				0.020**	
				(0.009)	
Above‐median income					−0.021***
					(0.008)
Economic Support Index (*ESI*)	−0.013	−0.014	−0.001	−0.011	−0.012
	(0.017)	(0.017)	(0.018)	(0.025)	(0.015)
*ESI interacted with*					
Female	0.006				
	(0.014)				
Tertiary education		0.010			
		(0.011)			
Partnered			−0.014		
			(0.011)		
Retired				0.002	
				(0.018)	
Key‐sector employee				−0.005	
				(0.018)	
Other‐sector employee				0.003	
				(0.020)	
Above‐median income					0.004
					(0.011)

*Note:* These are fixed‐effects regressions. The sample here is respondents from the four 2020 waves of the COME‐HERE survey; there are 20,337 observations in each column. The Stringency Index and the Economic Support Index are standardised over the estimation sample. Standard errors in parentheses are clustered at the individual level. All regressions control for dummies for current labor‐force status, and wave and individual fixed‐effects. All interactions refer to values of the interacted variables measured at Wave One. The reference category for labor‐force status is respondents who are not employed. *, **, and *** respectively indicate significance levels of 10%, 5% and 1%.

In column (1) of Table 3, the Stringency Index coefficient is 50 percent larger for women, consistent with lockdowns having increased the burden on women in terms of household chores (Alon *et al*., 2020; Farré *et al*., 2020) and that a household time‐allocation perceived by women as unfair reduces their subjective well‐being (Flèche *et al*., 2018, 2020).^9^ This is also in line with Pierce *et al*. (2020), where the prevalence of clinical levels of mental distress, as measured by the GHQ‐12, in the UK during 2020 rose faster for women than for men, and Adams‐Prassl *et al*. (2021), where the entire effect of lockdown on mental health in the US is driven by women. Using UK Understanding Society data, Etheridge and Spantig (2020) conclude that the reduced social capital caused by social distancing explains an important part of the gender gap in mental health observed in 2020. We find on the contrary no significant gender difference for the correlation with the Economic Support Index.

In columns (2) and (3) we uncover no heterogeneity with respect to education or partnership status. However, in column (4) both retirement and employment have a protective role against the negative consequences of stringency.^10^ This may reflect that over two‐thirds of individuals in the reference category here are women. All of these interactions are insignificant for the Economic Support Index in the bottom panel of Table 3.

Last, we check in column (5) whether the effects of pandemic policy differed across the household income distribution, as reflected by a dummy variable for the household having above‐median equivalised household income in January 2020. Our prior was that richer respondents probably had better resources to cope with stringent policies (via better housing and greater financial security, among others); however, the interaction term in column (5) is negative and significant. The richer half of our sample have rather suffered more from greater stringency than did the poorer half. This may reflect that stringency had a disproportionate effect on the types of leisure activities in which well‐off households are more likely to engage. Using semi‐nonparametric IV estimation of shape‐invariant Engel curves, Blundell *et al*. (2007) show that income increases food‐out expenditures. Income also has a positive correlation with the demand for tourism (Alegre and Pou, 2004) and cultural activities such as theatre (Ateca‐Amestoy, 2008), the access to both of which was sharply restricted or removed entirely by more stringent travel restriction and lockdowns.

## 4 Conclusion

We have used a unique relatively high‐frequency panel survey over 2020, covering France, Germany, Italy, Spain and Sweden, to show that more‐stringent policies significantly reduce life satisfaction. This correlation is found conditional on the spread of the pandemic itself. This fall in life satisfaction is larger for women, respondents with weak ties to the labor market, and those with relatively‐high household income. On the contrary, we find no life‐satisfaction effect of the economic support provided by governments, perhaps showing that this latter has served to compensate for the lower income that would have prevailed in its absence.

Our results have a number of policy implications. They first underline that lockdowns have had significant well‐being costs, which should enter the welfare calculus when determining pandemic policy. These costs are also not distributed equally: in particular, women’s well‐being has been more strongly affected. As well‐being is known to predict a number of behaviors, pandemic policy may not only have transient effects but also be reflected in future economic, social and political outcomes.

## Supporting information


**Table A1.** Predicting the Stringency Index by the Spread of COVID‐19
**Table A2.** Predicting the Economic Support Index by the Spread of COVID‐19
**Table A3.** Pandemic Policy and Life Satisfaction—Pooled and Panel Results (Full Results)
**Table A4.** Pandemic Policy and Life Satisfaction—Robustness Checks

## Footnotes

^1^
Stratification ensured that the data is representative in terms of gender, region, and age. Ethics approval was granted by the Ethics Review Panel of the University of Luxembourg.

^2^
For more details, see www.bsg.ox.ac.uk/research/research‐projects/coronavirus‐government‐response‐tracker#data.

^3^
We have also checked for anticipation effects. To do so, we use future values of the indices as independent variables in our life‐satisfaction regressions, controlling for the current index values at the interview date. None of these future values of the indices attracts significant estimates.

^4^
In alternative specifications, we introduced a linear time trend rather than wave fixed‐effects, and then omitted all time variables. In both cases, the significance levels are comparable and the estimated coefficients are somewhat larger. We use wave fixed‐effects in the main specification to account in a flexible way for the influence of confounders that are shared between individuals by wave.

^5^
The negative relationship between the Stringency Index and life satisfaction in COME‐HERE data is also found in Schifano *et al*. (2021). Their focus is rather on the correlation between subjective well‐being and working from home, and they control for the Stringency Index as a confounder of this correlation. They consider not only life satisfaction, but also a worthwhile life, loneliness, depression and anxiety. They do not examine the role of the Economic Support Index.

^6^
We also investigated a possible interaction between *SI* and *ESI* in the life‐satisfaction regressions. We found no evidence of this, either introducing the values of the indices in continuous form or as dummy variables for above/below their median values (by country).

^7^
Controlling for the parallel evolution of COVID‐19 itself using the seven other indicators in Tables A1 and A2 produces similar results: the Stringency Index reduces life satisfaction, and the Economic Support Index and COVID‐19 are statistically uncorrelated.

^8^
Our sample is smaller than that in some large‐scale social‐science panel surveys, such as the UK Household Longitudinal Study and the German Socio‐Economic Panel. This, in addition to our quite stringent regression specification, may limit our statistical power (in the sense that doubling our sample size would likely have made a number of our estimated coefficients, including that on mortality, significant. It is also possible that different individuals are sensitive to different measures of pandemic progress, so that there is not one best measure of COVID‐19 incidence.

^9^
We have checked whether this female penalty is stronger for mothers, the assumption being that having children increases the time spent in housework. Our results, available upon request, suggest the opposite: being a mother partially compensates for the female penalty in life satisfaction. This may be because children have a buffering effect against some of the negative consequences of stringency policies, such as loneliness.

^10^
The reference category in column (4) is individuals of working age who are without a job: the unemployed, homemakers and students. We do not find significant interaction terms when we consider these categories separately. Key‐sector employees are self‐identified from a question in the COME‐HERE survey asking whether the respondent worked in one of a list of “key sectors” as defined by many governments. Other‐sector employees are those who did not self‐identify as key‐sector workers.
