# Supplementary material for: Pandemic Policy and Life Satisfaction in Europe
Source: Rev Income Wealth. 2021 Oct 29;68(2):393–408. doi: 10.1111/roiw.12554 (PMC8661917; doi:10.1111/roiw.12554)
Supplement: Supplementary file 1 — Table A1. Predicting the Stringency Index by the Spread of COVID‐19 Table A2. Predicting the Economic Support Index by the Spread of COVID‐19 Table A3. Pandemic Policy and Life Satisfaction—Pooled and Panel Results (Full Results) Table A4. Pandemic Policy and Life Satisfaction—Robustness Checks [file ROIW-68-393-s001.docx]

**Online Appendix**

Table A1. Predicting the Stringency Index by the Spread of COVID-19

|  | Stringency Index (standardised) | | | | | | | |
| --- | --- | --- | --- | --- | --- | --- | --- | --- |
|  | (1) | (2) | (3) | (4) | (5) | (6) | (7) | (8) |
| Total No. Deaths | 0.263^***^ |  |  |  |  |  |  |  |
| (2-week average) | (0.057) |  |  |  |  |  |  |  |
|  |  |  |  |  |  |  |  |  |
| Total No. Deaths |  | 0.181^***^ |  |  |  |  |  |  |
| (4-week average) |  | (0.058) |  |  |  |  |  |  |
|  |  |  |  |  |  |  |  |  |
| Daily Deaths |  |  | 0.780^***^ |  |  |  |  |  |
| (2-week average) |  |  | (0.037) |  |  |  |  |  |
|  |  |  |  |  |  |  |  |  |
| Daily Deaths |  |  |  | 0.828^***^ |  |  |  |  |
| (4-week average) |  |  |  | (0.033) |  |  |  |  |
|  |  |  |  |  |  |  |  |  |
| Total No. Cases |  |  |  |  | 0.417^***^ |  |  |  |
| (2-week average) |  |  |  |  | (0.054) |  |  |  |
|  |  |  |  |  |  |  |  |  |
| Total No. Cases |  |  |  |  |  | 0.399^***^ |  |  |
| (4-week average) |  |  |  |  |  | (0.055) |  |  |
|  |  |  |  |  |  |  |  |  |
| Daily Cases |  |  |  |  |  |  | 0.355^***^ |  |
| (2-week average) |  |  |  |  |  |  | (0.056) |  |
|  |  |  |  |  |  |  |  |  |
| Daily Cases |  |  |  |  |  |  |  | 0.470^***^ |
| (4-week average) |  |  |  |  |  |  |  | (0.052) |
| R-squared | 0.069 | 0.033 | 0.608 | 0.686 | 0.174 | 0.159 | 0.126 | 0.221 |

*Notes*: These are OLS regressions of the value of the Stringency Index in Figure 2 on various measures of the spread of the pandemic. There are 285 observations in each column. Standard errors appear in parentheses.^*^ *p* < 0.1, ^**^ *p* < 0.05, ^***^ *p* < 0.01.

Table A2. Predicting the Economic Support Index by the Spread of COVID-19

|  | Economic Support Index (standardised) | | | | | | | |
| --- | --- | --- | --- | --- | --- | --- | --- | --- |
|  | (1) | (2) | (3) | (4) | (5) | (6) | (7) | (8) |
| Total No. Deaths | 0.554^***^ |  |  |  |  |  |  |  |
| (2-week average) | (0.049) |  |  |  |  |  |  |  |
|  |  |  |  |  |  |  |  |  |
| Total No. Deaths |  | 0.565^***^ |  |  |  |  |  |  |
| (4-week average) |  | (0.049) |  |  |  |  |  |  |
|  |  |  |  |  |  |  |  |  |
| Daily Deaths |  |  | 0.040 |  |  |  |  |  |
| (2-week average) |  |  | (0.059) |  |  |  |  |  |
|  |  |  |  |  |  |  |  |  |
| Daily Deaths |  |  |  | 0.106^*^ |  |  |  |  |
| (4-week average) |  |  |  | (0.059) |  |  |  |  |
|  |  |  |  |  |  |  |  |  |
| Total No. Cases |  |  |  |  | 0.138^**^ |  |  |  |
| (2-week average) |  |  |  |  | (0.059) |  |  |  |
|  |  |  |  |  |  |  |  |  |
| Total No. Cases |  |  |  |  |  | 0.148^**^ |  |  |
| (4-week average) |  |  |  |  |  | (0.059) |  |  |
|  |  |  |  |  |  |  |  |  |
| Daily Cases |  |  |  |  |  |  | -0.007 |  |
| (2-week average) |  |  |  |  |  |  | (0.059) |  |
|  |  |  |  |  |  |  |  |  |
| Daily Cases |  |  |  |  |  |  |  | 0.015 |
| (4-week average) |  |  |  |  |  |  |  | (0.059) |
| R-squared | 0.307 | 0.320 | 0.002 | 0.011 | 0.019 | 0.022 | 0.000 | 0.000 |

*Notes*: These are OLS regressions of the value of the Economic Support Index in Figure 2 on various measures of the spread of the pandemic. There are 285 observations in each column. Standard errors appear in parentheses.^*^ *p* < 0.1, ^**^ *p* < 0.05, ^***^ *p* < 0.01.

Table A3. Pandemic Policy and Life Satisfaction – Pooled and Panel Results (Full Results)

|  | Life Satisfaction (standardised) | | | | | |
| --- | --- | --- | --- | --- | --- | --- |
|  | (1) | (2) | (3) | (4) | (5) | (6) |
| Stringency Index | -0.056^***^ |  | -0.057^***^ | -0.051^**^ | -0.050^**^ | -0.046^**^ |
|  | (0.019) |  | (0.020) | (0.021) | (0.020) | (0.020) |
| Economic Support Index |  | 0.002 | -0.005 | -0.007 | -0.008 | -0.010 |
|  |  | (0.021) | (0.017) | (0.017) | (0.017) | (0.015) |
| Average Daily Deaths/100,000 inhabitants |  |  |  | -0.018 | -0.026 | -0.017 |
| (4-week average) |  |  |  | (0.017) | (0.017) | (0.014) |
| Female |  |  |  |  | -0.015 |  |
|  |  |  |  |  | (0.016) |  |
| Age |  |  |  |  | -0.026^***^ |  |
|  |  |  |  |  | (0.004) |  |
| Age-squared/100 |  |  |  |  | 0.035^***^ |  |
|  |  |  |  |  | (0.004) |  |
| Family size |  |  |  |  | 0.000 |  |
|  |  |  |  |  | (0.006) |  |
| Partnered |  |  |  |  | 0.154^***^ |  |
|  |  |  |  |  | (0.019) |  |
| Secondary education |  |  |  |  | -0.002 |  |
|  |  |  |  |  | (0.021) |  |
| Tertiary education |  |  |  |  | -0.063^***^ |  |
|  |  |  |  |  | (0.016) |  |
| Full-time employment |  |  |  |  | 0.095^***^ | 0.177^***^ |
|  |  |  |  |  | (0.023) | (0.051) |
| Part-time employment |  |  |  |  | 0.002 | 0.090^**^ |
|  |  |  |  |  | (0.034) | (0.036) |
| Marginal employment |  |  |  |  | -0.202^***^ | 0.071 |
|  |  |  |  |  | (0.076) | (0.061) |
| Log equivalent household income |  |  |  |  | 0.149^***^ |  |
| (January 2020) |  |  |  |  | (0.012) |  |
| *Wave and Country FE* | Yes | Yes | Yes | Yes | Yes | Yes |
| *Controls* | No | No | No | No | Yes | Yes |
| *Individual FE* | No | No | No | No | No | Yes |

*Notes*: These are linear regressions. The sample here is respondents from the four 2020 waves of the COME-HERE survey; there are 20337 observations in each column. The Stringency Index, Economic Support Index and average daily deaths variable are all standardised over the estimation sample. Standard errors in parentheses are clustered at the Stringency Index*Economic Support Index level. ^*^, ^**^, and ^***^ respectively indicate significance levels of 10%, 5% and 1%.

Table A4. Pandemic Policy and Life Satisfaction – Robustness Checks

|  | Life Satisfaction (standardised) | | | | | |
| --- | --- | --- | --- | --- | --- | --- |
|  | (1) | (2) | (3) | (4) | (5) | (6) |
| Stringency Index | -0.040^*^ |  |  |  |  |  |
| (day of interview) | (0.020) |  |  |  |  |  |
|  |  |  |  |  |  |  |
| Economic Support Index | -0.011 |  |  |  |  |  |
| (day of interview) | (0.015) |  |  |  |  |  |
|  |  |  |  |  |  |  |
| Stringency Index |  | -0.040^*^ |  |  |  |  |
| (1-week average) |  | (0.020) |  |  |  |  |
|  |  |  |  |  |  |  |
| Economic Support Index |  | -0.012 |  |  |  |  |
| (1-week average) |  | (0.015) |  |  |  |  |
|  |  |  |  |  |  |  |
| Stringency Index |  |  | -0.051^**^ |  |  |  |
| (3-week average) |  |  | (0.019) |  |  |  |
|  |  |  |  |  |  |  |
| Economic Support Index |  |  | -0.007 |  |  |  |
| (3-week average) |  |  | (0.014) |  |  |  |
|  |  |  |  |  |  |  |
| Stringency Index |  |  |  | -0.054^***^ |  |  |
| (4-week average) |  |  |  | (0.020) |  |  |
|  |  |  |  |  |  |  |
| Economic Support Index |  |  |  | 0.001 |  |  |
| (4-week average) |  |  |  | (0.013) |  |  |
|  |  |  |  |  |  |  |
| Stringency Index |  |  |  |  | -0.035^***^ | -0.044^***^ |
| (2-week average) |  |  |  |  | (0.010) | (0.010) |
|  |  |  |  |  |  |  |
| Economic Support Index |  |  |  |  | -0.001 | 0.009 |
| (2-week average) |  |  |  |  | (0.008) | (0.009) |

*Notes*: These are fixed-effects regressions. The sample here is respondents from the four 2020 waves of the COME-HERE survey; there are 20337 observations in each column. The Stringency Index and the Economic Support Index are standardised over the estimation sample. Standard errors in parentheses are clustered at the individual level. All regressions control for dummies for current labour-force status, and wave and individual fixed-effects. The reference category for labour-force status is respondents who are not employed at Wave One. Columns (1) to (4) consider different time periods for the measurement of the SI and ESI, while columns (5) and (6) address attrition in the panel data using cross-sectional and longitudinal weights respectively. ^*^, ^**^, and ^***^ respectively indicate significance levels of 10%, 5% and 1%.
